# Linc02527 promoted autophagy in Intrahepatic cholestasis of pregnancy

**DOI:** 10.1038/s41419-018-1013-z

**Published:** 2018-09-24

**Authors:** Jianguo Hu, Li Liu, Yangyang Gong, Lei Zhang, Xiaoling Gan, Xiaodong Luo, Tinghe Yu, Xiaocui Zhong, Xinru Deng, Lina Hu, Zhanyu Zhang, Xiaojing Dong

**Affiliations:** Department of Obstetrics and Gynecology, Second Affiliated Hospital, Chongqing Medical University, Chongqing, China

## Abstract

LncRNA plays a crucial role in human disease. However, the expression and function of LncRNA in ICP(Intrahepatic cholestasis of pregnancy) is still not fully elucidated. In this study, we found Linc02527 was increased expression in placenta and serum of ICP patients. Ectopically expression of Linc02527 promoted autophagy and proliferate in HTR8 cells. Silencing Linc02527 suppressed the autophagy and proliferate in HTR8 cells. Mechanically study revealed that Linc02527 regulated the expression of ATG5 and ATG7 by sponging miR-3185. Linc02527 directly binding to YBX1 and activated P21. The growth of C57 mouse was retarded when autophagy was activated. In normal condition, inhibited autophagy using chloroquine did not affect the growth of C57 mouse. However, in the condition of autophagy was activated, inhibited autophagy using chloroquine can improve the growth of C57 mouse. Overall, the results of this study identified Linc02527 as a candidate biomarker in ICP and a potential target for ICP therapy. Chloroquine was a potential drug for ICP therapy.

## Introduction

Intrahepatic cholestasis of pregnancy (ICP) is an obstetrical complication. It may lead to intrauterine fetal death. The hazard behind ICP is the excessive accumulation of bile acids in hepatocytes and the inhibition of bile excretion^1^. Overproduction of bile acids or their impaired removal from hepatocytes may be the reason of this pathology, which leads to increased bile acid serum concentration^1–4^.

Autophagy is an evolutionarily conserved process by which cytosolic components are sequestered into double-membrane vesicles and consequently fused with lysosomes^5,6^. Autophagy plays an important role in cellular pathways, including in growth, development and differentiation, aging and cell death^7–9^. Previous study reported that autophagy was involved in the progress of preeclampsia^10^. The marker of autophagy was abnormally expression in placenta with hypertensive disorders in pregnancy^11^. However, little is known about the function of autophagy in ICP.

LncRNA has been demonstrated that played an important role in human disease. LncRNA HOTAIR^12^, lncRNA MACC1-AS1^13^, LncRNA-MALAT1^14^ and LncRNA NEAT1^15^ were involved in cancer migration and invasion. In human placenta, some LncRNAs was vrefiied. Misregulation of LOC391533, LOC284100 and CEACAMP8 may contribute to the mechanism underlying preeclampsia^16^. lncRHOXF1 is the first lncRNA that regulates the host response to viral infections in human placental progenitor cells^17^. However, little is known about the expression and function of LncRNA in ICP.

In the present study, we investigated LncRNA expression and its potential clinical significance in ICP, trying to clarify the possible function of LncRNA in the pathogenesis.

## Materials and methods

### Tissue specimens

The placenta tissue and serum sample was were obtained from the second affiliated Department of Obstetrics and Gynecology, Second Affiliated Hospital, Chongqing Medical University. The Ethics Committee of the Chongqing Medical University approved the study documents and the use of archived placenta tissues and serum sample.

### Animal models

Thirty Sprague-Dawley pregnant rats were randomly divided into two groups: Group NP and Group ICP. The rats provided by animal experiment center of Chongqing medical university. The rats were maintained in collective cages in an appropriate room with controlled temperature and with a 12-h light cycle and fed with standard rat chow and water. They were aged at ~150–180 days and weighed ~250–270 g. From day 13 of gestation, rats in group ICP were injected intraperitoneally with estradiol benzoate (5 mg/kg, Abcam) for 7 days consecutively. Group NP (control group, named Group NP) was injected intraperitoneally with normal saline. On day 13 of gestation, the levels of TBA, alanine aminotransferase (ALT), aspartate aminotransferase (AST), and total bile acid(TBA) was detected in serum which was drawn from tail vein of pregnant rats. On day 21 of gestation, cesarean section was performed with anesthesia by intraperitoneal injection of 10% hydral with dosage of 3 mL/kg. The placenta was obtained for detecting the expression of LC3-II.

Forty C57 pregnant mice were randomly divided into four groups: group NC, group RAPA, group CQ and group CQ + RAPA. Mice were maintained in collective cages in an appropriate room with controlled temperature with a 12-h light cycle, and mice were allowed access to food and water ad libitum. From day 12 of gestation of gestation, mice in group NC were injected intraperitoneally with normal saline for 6 days consecutively. Group RAPA were injected intraperitoneally with rapamycin(5 mg/kg, CST). Group CQ were injected intraperitoneally with chloroquine (50 mg/kg, CST). Group RAPA + CQ were injected intraperitoneally with rapamycin(5 mg/kg, CST) and chloroquine (50 mg/kg, CST). On day 21 of gestation, the pregnant mice were sacrificed. Then, the fetal mice was calculated for body weight, fetus number, bodylenth and placenta weight.

### Microarray assay

Human LncRNA Microarray V4.0 (Agilent, USA) is designed for global profiling of human lncRNAs and protein-coding transcripts. Human miRNA Microarray Release 21.0 (Agilent technologies, USA) is designed for detecting the miRNA expression. The experiments and data analysis were performed by Beijing Boao Crystal Code Biotechnology Co. Ltd.

### Cell culture, transfection procedure, and reagents

Human HTR8 cells were cultured in RPMI 1640 medium (Sigma-Aldrich, R8758) containing 10% fetal bovineserum. The cells were incubated under 5% carbon dioxide at 37 °C. siRNA corresponding to the target sequences were synthesized by Genepharma (Shanghai, China). The following sequences were targeted for human Linc02527 small interfering RNA (siRNA): Linc02527-1: 5’-GGAGAGGCUAUAAACUUCUTT-3′; Linc02527-2: 5′-CCGUGCAUAUUGUCUCCAATT-3′; and NC (negative control) siRNA: 5′-UUCUUCGAAGGUGUCACGUTT-3′. Lentiviral vectors expressing shRNA targeting Linc02527 (named LV3-lnc02527-1 and LV3-lnc02527-2) and the Linc02527-lentiviral expression vector (named LV5-lnc02527) were provided by Genepharma. miR-3185 mimics (sense: 5′-AGAAGAAGGCGGUCGGUCUGCGG-3′) were synthesized at Ruibo Biotechnology(Guangzhou, China). AST detecting using AST Activity Assay Kit(MAK055, Sigma), ALT detecting using ALT Activity Assay(MAK052, Sigma); TBA detecting using Total Bile Acids (TBA) Assay Kit (Colorimetric) (BioVision.Inc, America); CG detecting using CG RIA Kit(YB-10016, Shanghai Zibo Biotechnology Co., Ltd. China).

### Flow cytometric analysis of cell apoptosis

Cells for apoptotic analysis were double stained with Annexin V–FITC and propidium iodide 48 h after transfection and analyzed using a flow cytometer (FACScan; BD Biosciences, Shanghai, China) equipped with CellQuest software (BD Biosciences). Each experiment was performed in triplicate.

### RNA extraction and Quantitative real-time polymerase chain reaction(RT-qPCR)

Total RNA was isolated using a pure high-purity Total RNA Rapid Extraction Kit (Bioteke Corporation, RP1201) according to the manufacturer’s instruction. cDNA was synthesized using the iSCRIPT cDNA synthesis kit (Bio-Rad Laboratories, 4106228). All of the primers used to amplify lncRNAs were synthesized by GenePharma. The real-time PCR kit was purchased from GeneCopoeia. PCR conditions were 95 °C for 10 s, 60 °C for 20 s and 72 °C for 10 s. Each sample was analyzed in triplicate. Relative quantification of mRNA was performed using the comparative threshold cycle (CT) method. This value was used to plot the gene expression using the formula 2^−Δ ΔCT^.

### Dual-luciferase reporter gene assay

Luciferase reporter gene assay was performed using the Dual-Luciferase Reporter Assay System (Promega Corporation, E1910) according to the manufacturer’s instruction. Luciferase reporter assays for ATG5, ATG7 and Linc02527, wild-type or mutant reporter constructs (termed WT or Mut; obtained from Genepharma) were cotransfected into HTR8 cells in 24-well plates with 100 nM MIR3185 or 100 nM miR-NC and Renilla plasmid by using Endofectin-Plus (Gene-Copoeia, Z01010A). All experiments were performed at least 3 times.

### Western blotting

The expressions of LC3, ATG7, ATG5, YBX1, P21 and actin proteins were analyzed by western blot^18,19^. The primary antibodies used include polyclonal rabbit anti- LC3 (Cell Signaling Technology, 4108); rabbit monoclonal to ATG5 (Abcam, ab109490); rabbit monoclonal to ATG7(Abcam, ab183188); rabbit monoclonal to YBX1 (Abcam, ab76149); rabbit monoclonal to P21 (Abcam, ab109520); and monoclonal rabbit anti-beta actin (Abcam, ab115777). The band density was analyzed using a gel imaging system and compared with an internal control.

### Cell proliferation assay

Cell proliferation was determined using the EdU assay. It was performed using the Cell-Light TM EdU imaging detecting kit according to the instructions in the kit (Ruibo Biotechnology, Guangzhou, China)^20,21^.

### RNA pulldown assay and matrix-assisted laser desorption/ ionization time of flight mass spectrometry (MALDI-TOF-MS)

To find the proteins bonding to Linc02527 directly, the RNA pull-down assay was performed^22,23^. The specific operation steps was according to the previous study^24^. Using SDS-PAGE gel electrophoresis, Silver stain and MALDI-TOF-MS, then, the specific proteins were identified.

### Statistical analysis

All statistical analyses were performed using SPSS software, version 17.0 (Chicago, IL). Each experiment was performed in triplicate. Statistical analysis was performed by Student t test. Pearson correlation analysis was conducted to analyze the association between Linc02527 expression and serum TBA, CG, ALT or AST. Data were presented as mean ± standard deviation. Statistical significance was defined as a p-value less than 0.05.

## Results

### Microarray expression profiles of lncRNAs and mRNAs in placenta of ICP patients

**Fig. 1:**
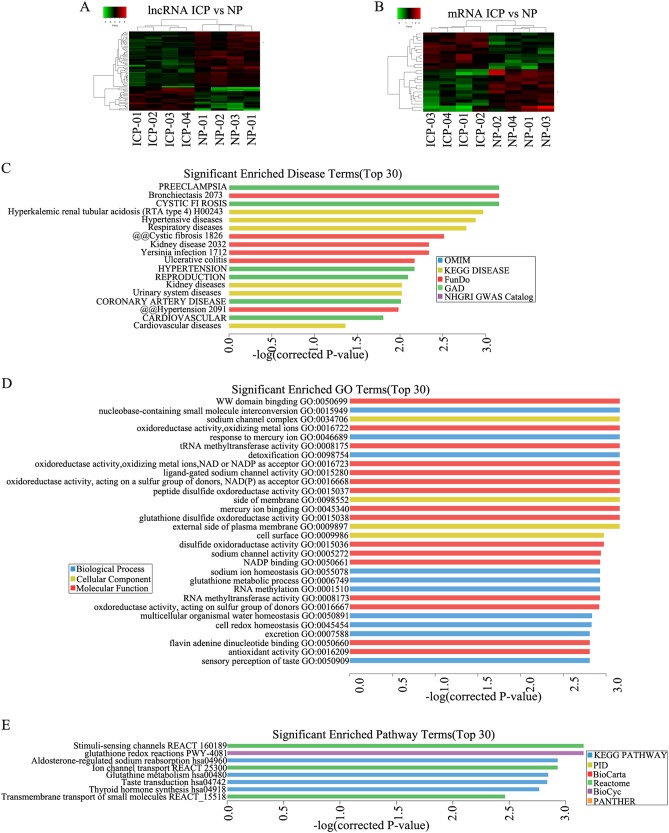
Hierarchical clustering of **a** lncRNA and **b** mRNA differential expression profiles between the ICP group and control group(NP) in 8 placenta samples. The heat maps are based on expression values of significantly differentially expressed lncRNAs and mRNAs (absolute fold change ≥1.5 and *P* < 0.05) detected by microarray probes. “Red” and “Green” indicated expression above and below, respectively, relative expression. **c** Terms enriched disease-related LncRNAs(top 30). **d** Go and Kegg (**e**) pathway analysis of differentially expressed LncRNAs(top 30)

To explore the potential biological functions of lncRNAs in ICP, we examined the expression patterns of lncRNAs and mRNAs in placenta of ICP patients and controls with full term pregnancy. In total, 358 lncRNAs and 255 mRNAs showed a ≥1.5-fold change (*P* < 0.05) in 8 samples (Fig. 1a, b). Of these, 100 lncRNAs were up-regulated, and 258 were down-regulated; 123 mRNAs were up-regulated, 132 were down-regulated (Table 1 and S1). The differential expression lncRNAs were related to respiratory diseases, hypertensive diseases, cardiovascular diseases and so on (Fig. 1c). These lncRNAs were involved in many cellular process including in sodium channel activity, peptide disulfide oxdoreductase activity and so on (Fig. 1d). They also participated in aldosterone-regulated sodium reabsorption, Stimuli-sensing channels, glutathine metabolism and transmembrane transport of small molecules (Fig. 1e).

**Table 1:** Summary of lncRNA that are differently expressed between placenta from NP and ICP (ICP vs. NP)

Gene symbol	Chromosomal location	Regulation	*P*-value
LINC02027	Chr3:810430188-81144798(+)	Up	0.02699606
LINC02527	Chr6:112221691-112223054(−)	Up	0.01485026
FAM239A	ChrX: 3771050-3781615(−)	Up	0.025825039
KCNMB2-AS1	Chr3:178447071-178577338(−)	Up	0.028023811
YEATS2-AS1	Chr3:183524244-183526729(−)	Up	0.0014545523
LINC02050	Chr3: 80814047-80838505(+)	Up	0.09413093
TCONS_00005551	Chr3:81143793-81144462(+)	Up	0.02998128
TCONS_00011228	Chr6:112221561-112230600(−)	Up	0.014197185
LINC01436	Chr21: 36005338-36007838(+)	Up	0.010051289
HIT000075265	Chr16:23315293-23360088(+)	Down	0.021368366
uc021wnb.1	Chr22: 24245625-24247864(−)	Down	0.024611032

### Linc02527 expression was increased in placenta and serum of ICP

**Fig. 2:**
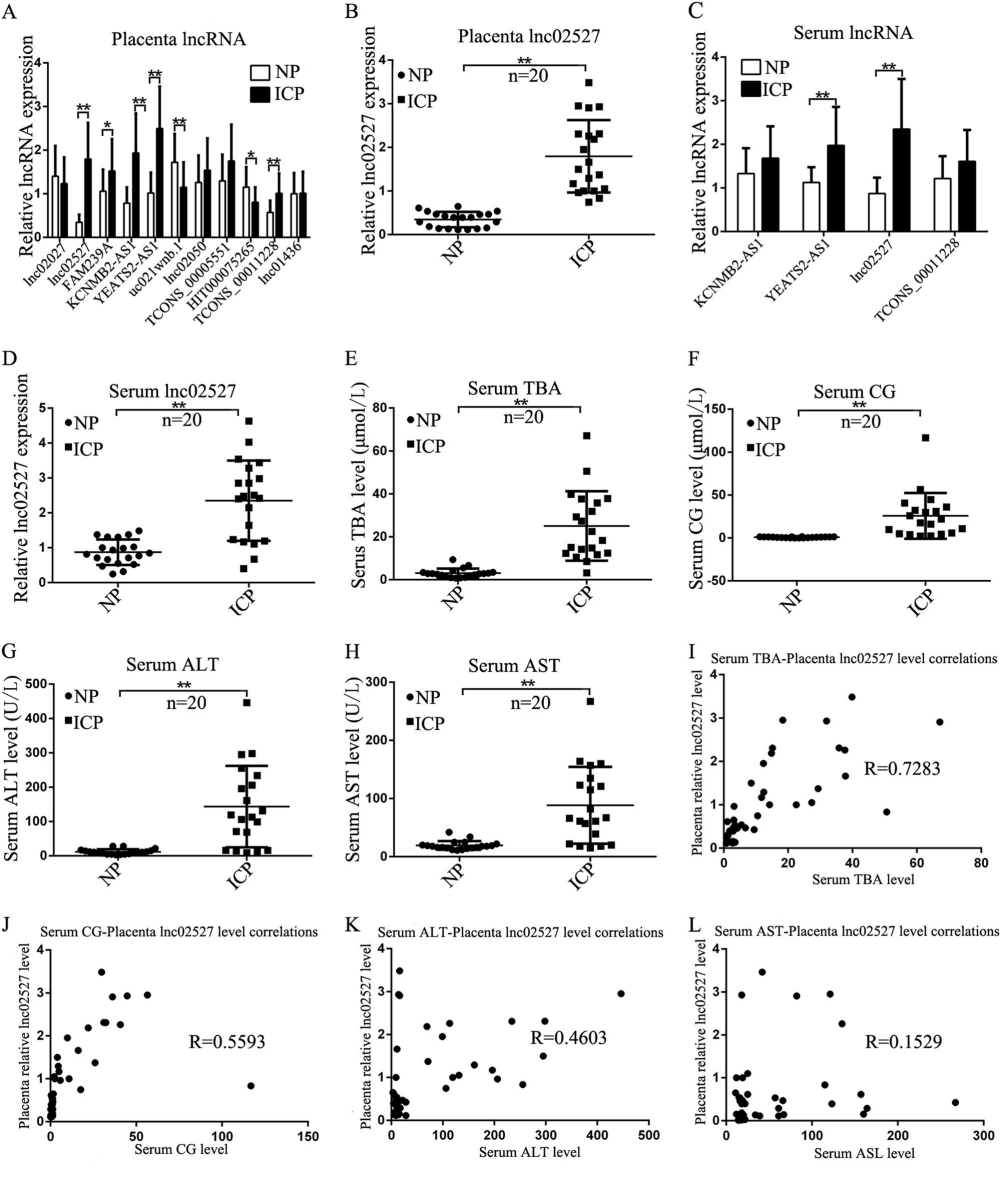
Linc02527 was detected in placenta and serum of ICP. **a** The LncRNA was validated by qPCR in placenta of ICP (*n* = 20) and normal control (*n* = 20). **b** Linc02527 was detected in placenta of ICP (*n* = 20) and normal control (*n* = 20). **c**, **d** The expression of LncRNA was detected in serum of ICP (*n* = 20) and normal control (*n* = 20). **e**–**h** The serum TBA, CG, ALT and AST were detected in ICP patients (ICP; *n* = 20) and normal control (NP; *n* = 20). **i**–**l** The expression of Linc02527 in placenta was positively correlated with the expression of serum TBA, CG, ALT and AST. Error bars represented standard error. Asterisks(*) and ** indicated *p* < 0.05 and 0.001, respectively

We verified the expression of 11 LncRNA in placenta of ICP. We found that Linc02527, KCNMB2-AS1, YEATS2-AS1 and TCONS-00011228 were significant increased in placenta of ICP (Fig. 2a, b, Fig. 2S1). We also observed Linc02527, KCNMB2-AS1, YEATS2-AS1 and TCONS-00011228 expression was significantly increased in serum of ICP (Fig. 2c, d and Fig. S2A-C). The serum TBA, CG, ALT and AST was increased in ICP compared with that normal pregnant (Fig. 2e–h and Table 2). The Birth body length of newborn in ICP group was shorter than that of normal group (Table 3). The serum TBA, CG, ALT and AST was positively correlated with the expression of Linc02527 in placenta (Fig. 2i–l). The serum TBA, CG, ALT and AST was positively correlated with the expression of Linc02527 in serum (Fig. 2 S2D-G).

**Table 2:** Biochemistry (mean ± SD)

Parameter	NP (*n* = 20)	ICP (*n* = 20)	*P* value
TBA (µmol/L)	3.06 ± 2.12	25.04 ± 16.19	<0.01
CG (µmol/L)	0.85 ± 0.49	25.71 ± 26.66	<0.01
ALT (U/L)	11.75 ± 7.30	143.25 ± 118.48	<0.01
AST (U/L)	19.35 ± 7.55	88.4 ± 66.05	<0.01
Total bilirubin (µmol/L)	9.10 ± 2.62	11.64 ± 5.39	NS
Direct bilirubin (µmol/L)	3.11 ± 1.49	4.92 ± 3.64	<0.05
Indirect bilirubin (µmol/L)	6.00 ± 2.19	6.72 ± 3.15	NS

**Table 3:** Subject demographic and characteristics (mean ± SD)

Parameter	NP (*n* = 20)	ICP (*n* = 20)	*P* value
Age (years)	30.95 ± 3.35	29.20 ± 4.34	NS
Height (cm)	160.45 ± 4.84	159.70 ± 2.68	NS
Weight (kg)	70.325 ± 7.87	67.25 ± 8.19	NS
Weight gain (kg)	14.275 ± 4.31	13.625 ± 2.86	NS
Gravidity	2.30 ± 1.78	2.45 ± 1.76	NS
Parity	1.25 ± 0.44	1.3 ± 0.47	NS
Gestational weeks at delivery	38.39 ± 0.95	37.82 ± 1.03	NS
Birthweight (kg)	3.2 ± 0.32	3.04 ± 0.44	NS
Birth body length (cm)	50.35 ± 1.27	48.85 ± 1.90	<0.01

### Linc02527 promoted proliferation and autophagy in HTR8 cells

**Fig. 3:**
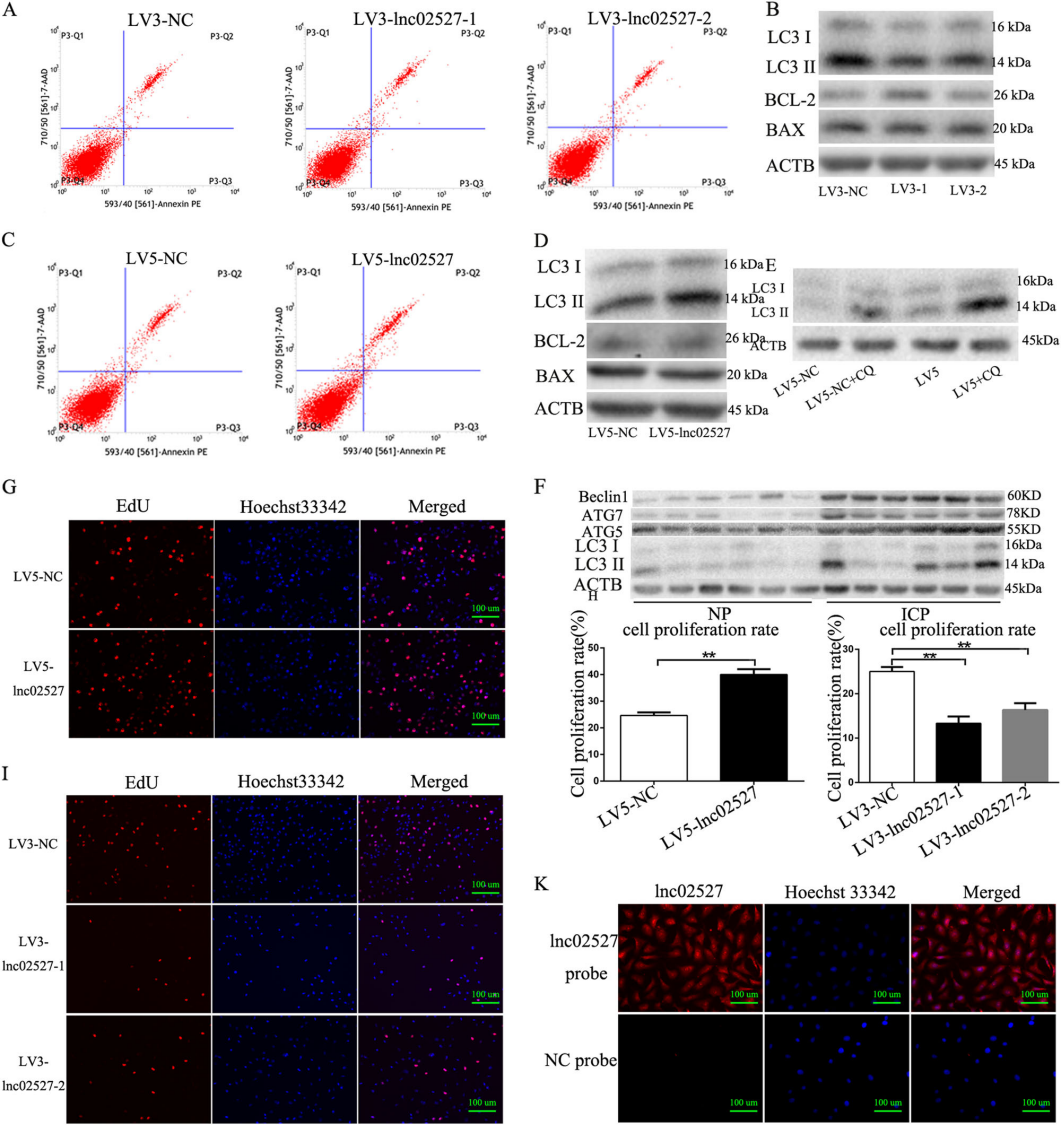
Linc02527 regulated autophagy and proliferate in HTR8 cells. **a** The apoptosis rate was detected using flow cytometry when silencing Linc02527. **b** The expression of LC3-II, BCL-2 and BAX was detected by western blot after silencing Linc02527.(**c**)The apoptosis rate was detected using flow cytometry when ectopically expression of Linc02527. **d** The expression of LC3-II, BCL-2 and BAX was detected by western blot when ectopically expression of Linc02527. **e** The expression of LC3-II was detected by western blot. LV5-NC: HTR8 cells infected by LV5-NC. LV5-NC + CQ: HTR8 cells infected by LV5-NC, then adding chloroquine at 10uM for 24 h. LV5: HTR8 cells infected by LV5-lnc02527. LV5 + CQ: HTR8 cells infected by LV5- lnc02527, then adding chloroquine at 10 uM for 24 h. **f** The expression of LC3 in placenta of ICP and normal group was detected by western blot. **g**–**j** The cellular proliferation was detected by EdU. **k** The location of Linc02527 was determined hybridization in situ. Lnc02527 probe was detected the location of Linc02527. NC was negative control. Error bars represented standard error. Asterisks(*) and ** indicated *p* < 0.05 and 0.001, respectively

We next determined the function of Linc02527. We firstly detected the apoptosis rate after silencing or ectopically expressed Linc02527 in HTR8 cells. The apoptosis rate is not significant difference between HTR8 cells infected with LV3-lnc02527-1 or lnc02527-2 and HTR8 cells infected with LV3-NC (Fig. 3a). Also, the apoptosis rate is not changed in HTR8 cells infected with LV5-lnc02527 compared with infected with LV5-NC (Fig. 3c). The expression of BCL-2 and Bax was not changed when silencing or ectopically expressed Linc02527 in HTR8 cells (Fig. 3b, d). This data revealed that Linc02527 did not regulate apoptosis in HTR8 cells. So we detected whether Linc02527 regulated autophagy in HTR8 cells. As we expected, the expression of LC3-II was decreased in HTR8 cells infected LV3-lnc02527-1 or lnc02527-2 compared to that infected LV3-NC (Fig. 3b). The expression of LC3-II was increased in HTR8 cells infected LV5-lnc02527 compared to that infected LV5-NC (Fig. 3d).

To investigate whether Linc02527 regulated autophagic flux, we quantified the levels of LC3-II, the autophagosome associated form of the protein, in the absence and presence of the lysosomal inhibitor chloroquine. The ratio between the LC3-II levels with and without chloroquine as an index of overall autophagic flux^25^. We observed a increase of this ratio in HTR8 cells infected LV5-lnc02527 compared to that infected LV5-NC (Fig. 3e).This data indicated that Linc02527 promoted autophagic flux. We determined whether autophagy was activated in the placenta of ICP. We found that the expression of LC3-II, ATG5, ATG7 and Beclin1 was increased in placenta of ICP compared to normal pregnant (Fig. 3f).

We also investigated whether Linc02527 was involved in the proliferate ability. We observed that silencing Linc02527 suppressed cellular proliferate; however, ectopically expressed Linc02527 promoted the cellular proliferate ability in HTR8 cells (Fig. 3g–i). To determine the mechanism of Linc02527, the location of Linc02527 was detected. Our results revealed that Linc02527 was located in the cytoplasm and nucleus (Fig. 3k).

### Linc02527 promoted autophagy by sponging miR-3185

**Fig. 4:**
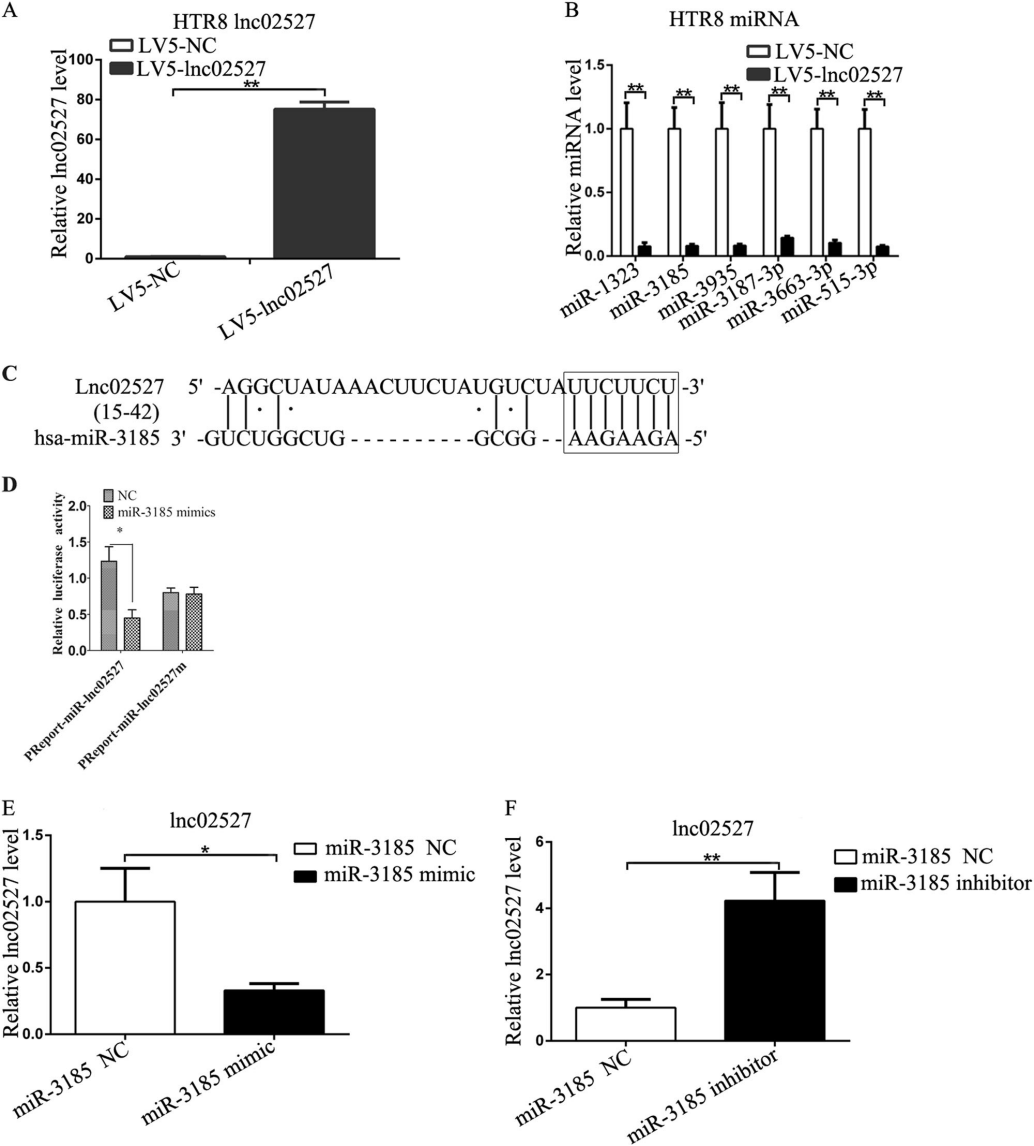
The expression of miR-3185 was regulated by Linc02527. **a** The expression of Linc02527 was determined by qPCR. **b** The miRNAs expression was detected by qPCR in HTR8 cells infected LV5-lnc02527 or LV5-NC. **c** The potential binding sites between Linc02527 and miR-3185 were predicted. **d** HTR8 cells were co-transfected with miR-3185 mimic or control RNA (NC) with luciferase reporter plasmids containing either wild-type (pMIR-lnc02527) or mutant (pMIR-lnc02527m) of Linc02527 genes. Luciferase expression was measured. The fold changes of the relative luciferase activity in miR-3185 mimic indicated that plasmid transfected cells were normalized to NC, indicating plasmid transfected cells. **e** The expression of Linc02527 was detected by transfecting miR-3185 mimic or NC(negative control). **f** The expression of Linc02527 was detected by transfecting miR-3185 inhibitor or NC(negative control). Error bars represented standard error. Asterisks(*) and ** indicated *p* < 0.05 and 0.001, respectively

The expression of Linc02527 was increased in HTR8 cells infected LV5-lnc02527 compared to that infected LV5-NC (Fig. 4a). The differential miRNA expression was screened using miRNA array (S2). We verified six most significant down-regulated genes using qPCR. Six miRNAs (miR-1323, miR-3185, miR-3935, miR-3187, miR-3663 and miR-515) was decreased in HTR8 cells infected LV5-lnc02527 compared to that infected LV5-NC (Fig. 4b and S2). Bioinformatics prediction (LncBase Predicted v.2) that there was a binding site between miR-3185 and Linc02527 (Fig. 4c). Luciferase reporter gene assay confirmed the binding of miR-3185 with Linc02527 (Fig. 4d). Over-expression of miR-3185 suppressed the expression of Linc02527 (Fig. 4e). However, silencing miR-3185 increased the expression of Linc02527(Fig. 4f).

**Fig. 5:**
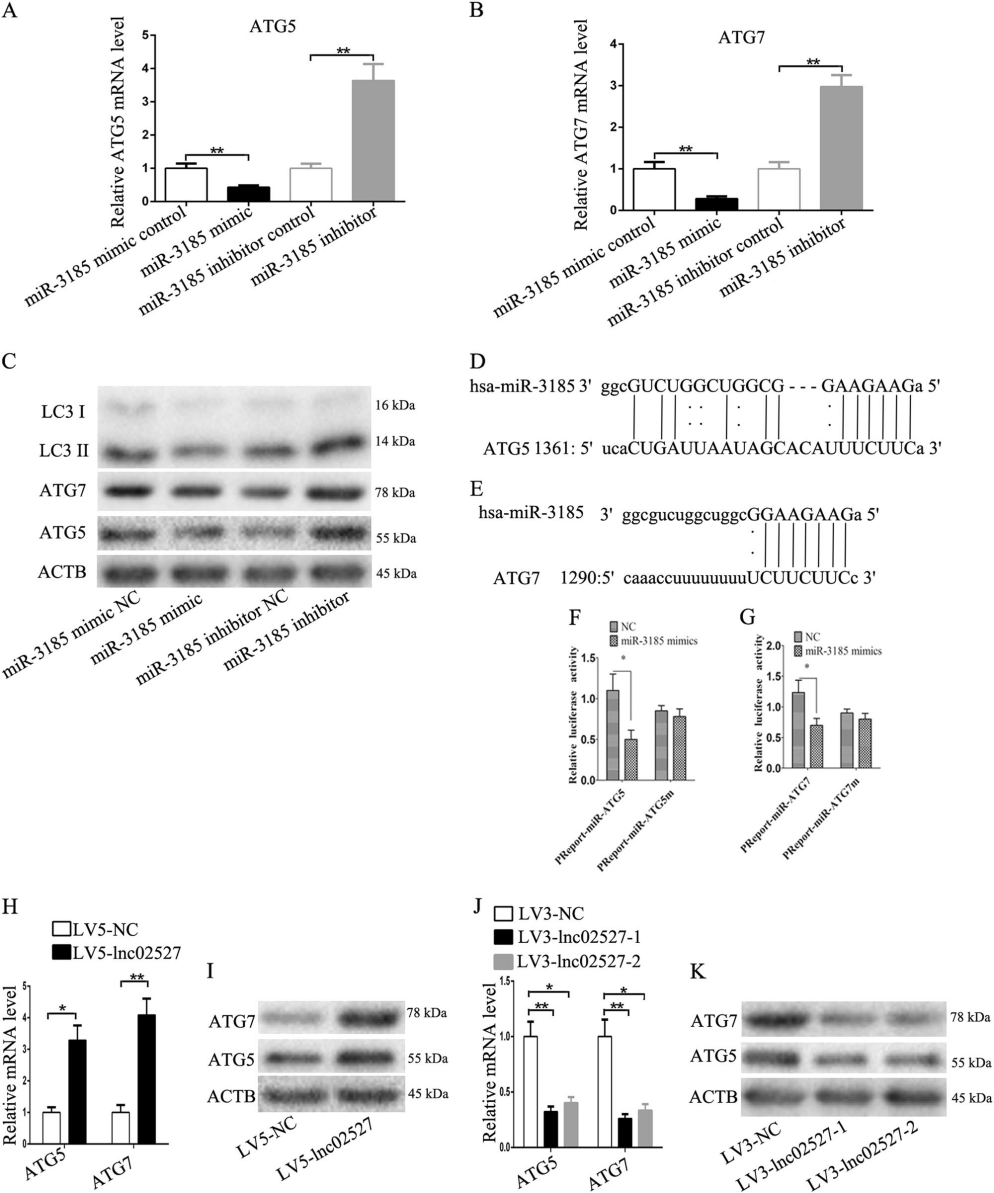
The expression of ATG5 and ATG7 was regulated by miR-3185. **a** The expression of ATG5 mRNA was detected when transfecting with miR-3185 mimic, miR-3185 mimic control, miR-3185 inhibitor or miR-3185 inhibitor mimic. **b** The expression of ATG7 mRNA was detected when transfecting with miR-3185 mimic, miR-3185 mimic control, miR-3185 inhibitor or miR-3185 inhibitor mimic. **c** The protein expression of ATG5, ATG7 and LC3-II was determined when transfecting with miR-3185 mimic, miR-3185 mimic control, miR-3185 inhibitor or miR-3185 inhibitor mimic. **d**, **e** The potential binding sites between miR-3185, ATG5 and ATG7 were predicted. **f**, **g** HTR8 cells were co-transfected with miR-3185 mimic or control RNA (NC) with luciferase reporter plasmids containing either wild-type (pMIR-ATG5-3’UTR and pMIR-ATG7-3’UTR) or mutant (pMIR-ATG5-3’UTRm and pMIR-ATG7-3’UTRm) of ATG5 and ATG7 genes. Luciferase expression was measured. The fold changes of the relative luciferase activity in miR-3185 mimic indicated that plasmid transfected cells were normalized to NC, indicating plasmid transfected cells. **h**, **i** The expression of ATG5 and ATG7 was determined in HTR8 cells infected with LV5-NC or LV5-lnc02527. **j**, **k** The expression of ATG5 and ATG7 was determined in HTR8 cells infected with LV3-NC, LV2-lnc02527-1 or LV2-lnc02527-2. Error bars represented standard error. Asterisks(*) and **indicated *p* < 0.05 and 0.001, respectively

Bioinformatics prediction(microRNA.org) that ATG5 and ATG7 were the potential target of miR-3185. So we detected the expression of ATG5 and ATG7 through silencing or over-expression of miR-3185 in HTR8 cells. The expression of ATG5 and ATG7 was decreased after over-expression of miR-3185. In contrast, the expression of ATG5 and ATG7 was increased after silencing miR-3185 (Fig. 5a–c). The LC3-II also was regulated by miR-3185 (Fig. 5c). We predicted that there was a binding site between miR-3185, ATG5 and ATG7 (Fig. 5d, e). Luciferase reporter gene assay confirmed the binding of miR-3185 with ATG5 and ATG7 (Fig. 5f, g). The expression of ATG5 and ATG7 was increased in HTR8 cells infected LV5-lnc02527 compared to that infected LV5-NC (Fig. 5h, i). The expression of ATG5 and ATG7 was decreased in HTR8 cells infected LV3-lnc02527-1 or LV3-lnc02527-2 compared to that infected LV3-NC (Fig. 5j, k). This data suggested that Linc02527 promoted autophagy by sponging miR-3185.

### Linc02527 directly binding to YBX1 and activated P21

**Fig. 6:**
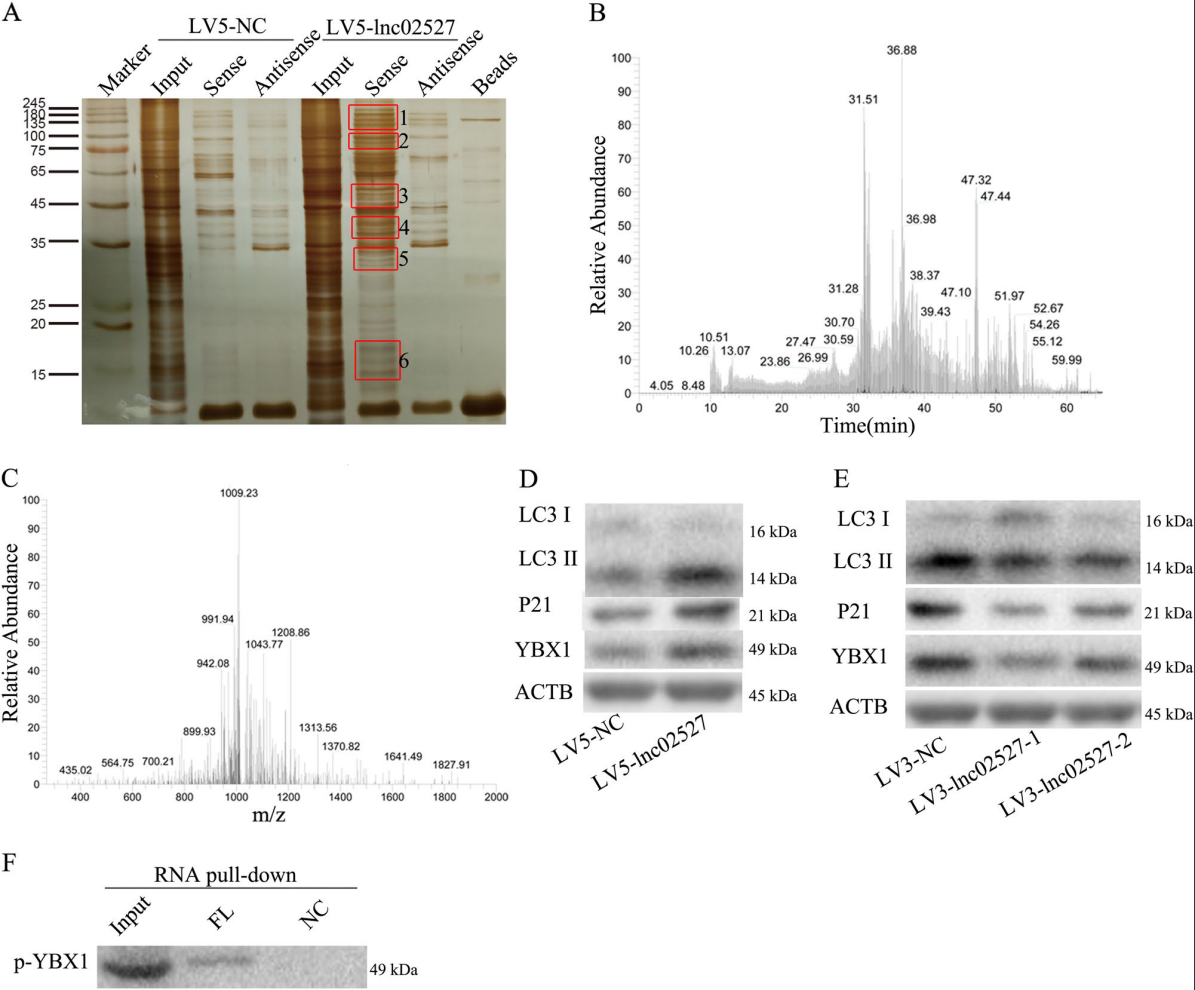
Linc02527 directly binding to YBX1 and activated P21. **a** Silver stains for protein gels obtained by Linc02527 RNA Pulldown. Bio-lnc02527 (sense): treatment group; Bio-lnc02527 (antisense). The Linc02527 specific binding protein gels (in red box) was identified by MALDI-TOF-MS. Results show that YBX1 was a direct binding protein to Linc02527. **b**, **c** YBX1 was identified by MALDI-TOF-MS. **d** The expression of LC3-II,YBX1 and P21 was determined in HTR8 cells infected with LV5-NC or LV5-lnc02527. **e** The expression of LC3-II,YBX1 and P21 was determined in HTR8 cells infected with LV3-NC, LV2-lnc02527-1 or LV2-lnc02527-2. **f** The Linc02527 specific binding protein gels was identified by RNA pulldown. FL was the biotin Linc02527. NC was the Linc02527 without biotin label

To further explore the mechanism of Linc02527 regulation autophagy. The RNA pull down assay was performed. Then using SDS-PAGE gel electrophoresis and Silver stain six differential bands were obtained(Fig. 6a). Using MALDI-TOF-MS, some protein including YBX1 were identified(S3). It indicated that YBX1 was directly bond to Linc02527(Fig. 6a–c).We confirmed that YBX1 was directly bond to Linc02527 through RNA pull down assay(Fig. 6f). The expression of YBX1 was increased in HTR8 cells infected LV5-lnc02527 compared to that infected LV5-NC(Fig. 6d). The expression of YBX1 was decreased in HTR8 cells infected LV3-lnc02527-1 or LV3-lnc02527-2 compared to that infected LV3-NC(Fig. 6e). Previous study reported that P21 pathway was activated by YBX1^26^. P21 pathway can promotes autophagy^27–29^. P21 was elevated when over-expression of Linc02527. P21 was down-regulated when silencing Linc02527 expression(Fig. 6d, e). So, we guessed Linc02527 bond to and stabilized YBX1, then activated P21 pathway and prompted autophagy.

### Autophagy was involved in growth of fetal mouse

**Fig. 7:**
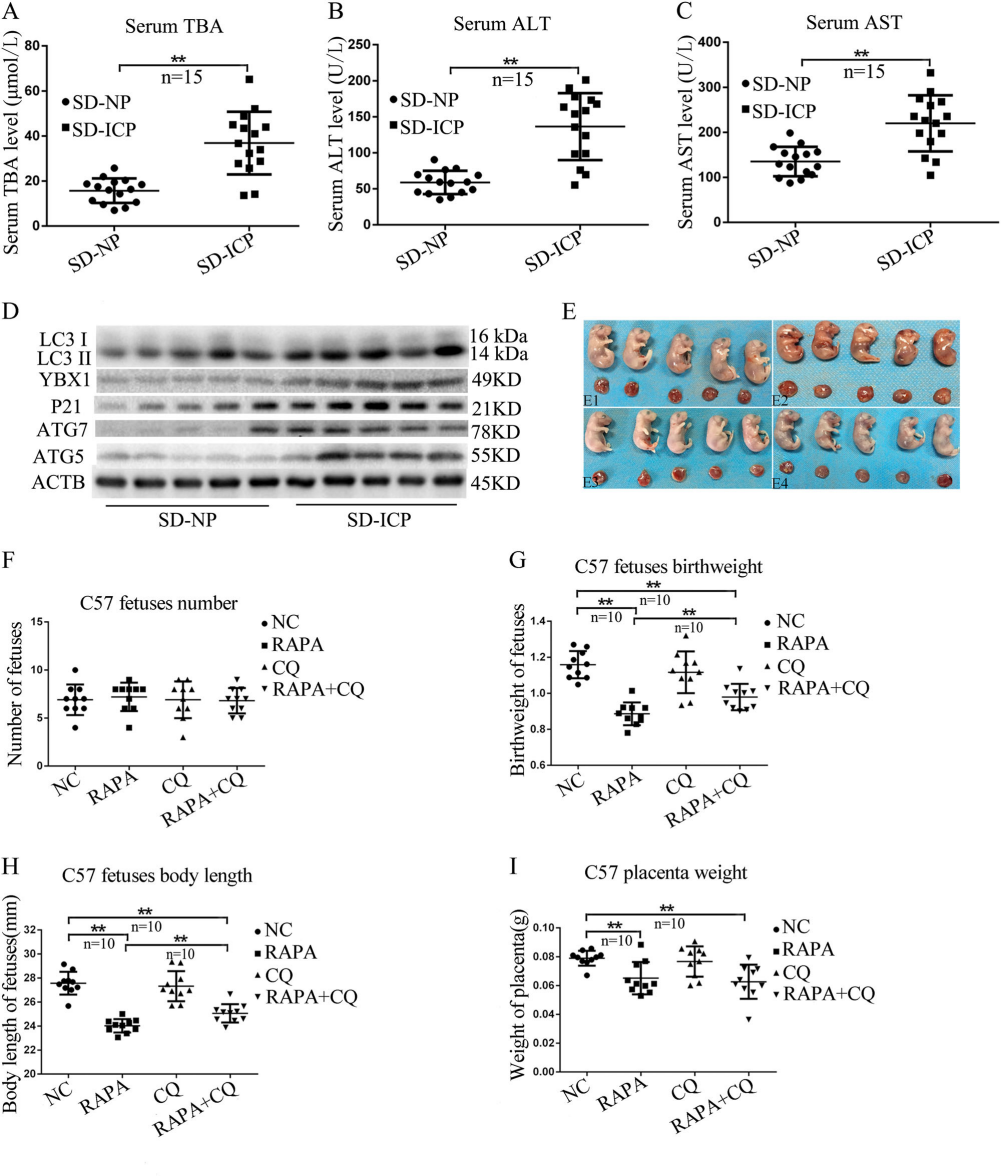
Autophagy was involved in growth of fetal mouse. **a**–**c** The serum TBA, ALT and AST were detected in in ICP in the rat model. **d** The expression of LC3-II was detected in the placenta of ICP rat or NP group(normal control). **e**–**i** Forty C57 pregnant mice were randomly divided into four groups: group NC(E1), group RAPA(E2), group CQ(E3) and group CQ + RAPA(E4). From day 12 of gestation of gestation, mice in group NC were injected intraperitoneally with normal saline for 6 days consecutively. Group RAPA were injected intraperitoneally with rapamycin (5 mg/kg). Group CQ were injected intraperitoneally with chloroquine (50 mg/kg). Group RAPA + CQ were injected intraperitoneally with rapamycin (5 mg/kg) and chloroquine (50 mg/kg). On day 21 of gestation, the pregnant mice were sacrificed. Then, the fetal mice was calculated for body weight, fetus number, bodylenth and placenta weight. Error bars represented standard error. Asterisks(*) and **indicated *p* < 0.05 and 0.001, respectively

We have demonstrated Linc02527 promoted autophagy in vitro. However, we did not find homologous sequence of Linc02527 in rat and mouse. We observed autophagy was activated in ICP. So, we detected whether autophagy was activated in ICP in the rat model. The serum TBA, ALT and AST was increased in ICP in the rat model (Fig. 7a–c). The LC3-II, ATG5 and ATG7 expression was elevated in the placenta of ICP rat compared to normal group (Fig. 7d). This data revealed that autophagy was activated in ICP rat. We also observed YBX1 and P21 was elevated in the placenta of ICP rat compared to normal group (Fig. 7d). Then, we explore whether activated autophagy or inhibited autophagy can affect the growth of C57 mouse. We observed that the growth of C57 mouse was retarded when autophagy was activated using rapamycin compared to normal group (Fig. 7E1–2). Compared with the normal group, the growth of C57 mouse is not different when autophagy was inhibited using chloroquine (Fig. 7E3). The inhibition of growth by rapamycin is partly relieved through adding chloroquine (Fig. 7E4). The number of fetuses is no significant difference between normal control group, rapamycin group, chloroquine group and rapamycin + chloroquine group (Fig. 7f).The fetuses birth weight, body length and placenta weight were decreased in rapamycin group than that of normal control group (Fig. 7g–i). The fetuses birth weight, body length and placenta weight were decreased in rapamycin group than that of normal control group (Fig. 7g–i). The fetuses birth weight, body length and placenta weight were decreased in rapamycin + chloroquine group than that of rapamycin group (Fig. 7g–i). There was not significant difference between chloroquine group and normal control group (Fig. 7g–i). These data revealed that the growth of C57 mouse was repressed when autophagy was activated. In normal condition, inhibited autophagy using chloroquine did not affect the growth of C57 mouse. However, in the condition of autophagy was activated, inhibited autophagy using chloroquine can improve the growth of C57 mouse. Rapamycin caused fetal growth retardation, possibly due to inhibition of the mTOR pathway.

## Discussion

In the present research, we found that Linc02527 was increased expression in serum and placenta of ICP patients. Linc02527 promoted autophagy in HTR8 cells. Linc02527 regulated ATG5 and ATG7 though sponging miR-3185. Linc02527 directly binding to YBX1 and activated P21, then the autophagy was promoted. We also found that regulation of autophagy was mediated the growth of fetal mouse.

Linc02527 was located in 6q21. In 27 different human tissues, it is distinctive high-expression in placenta and testis^30^. In this study, we observed Linc02527 expression was increased in the placenta and serum of ICP patients. The serum TBA, CG, ALT and AST was elevated in ICP patients. The serum TBA and CG were diagnostic criteria of ICP^31–33^. The expression of Linc02527 was positively with the serum TBA, CG, ALT and AST. So, Linc02527 is a potential biomarker of Intrahepatic Cholestasis of Pregnancy(ICP).

In this research, we noted that Linc02527 was not involved in apoptosis. However, it promoted autophagy and proliferate. Many studies have demonstrated that LncRNA regulated the target genes by sponging miRNAs^34–36^. ATG5 and ATG7 are two important moleculars to promote autophagy^37–41^. We found miR-3185 inhibited autophagy by targeting ATG5 and ATG7. Linc02527 directly bond to and down-regulated miR-3185. Our data revealed Linc02527 regulated ATG5 and ATG7 through sponging miR-3185. YBX1 regulates tumor growth via CDC25a pathway in human lung cancer^42^. lncRNA GAS5 enhances G1 cell cycle arrest via binding to YBX1 to regulate p21 expression in stomach cancer^26^. Phosphorylation of YBX1 activates NF-κB in colon cancer. We found Linc02527 directly bond to and stabilized YBX1. At the same time, Linc02527 increased the expression of P21. P21 pathway can promotes autophagy^27–29^. So, we guessed Linc02527 bond to and stabilized YBX1, then activated P21 pathway and prompted autophagy. These results indicated that Linc02527 was a potential target for ICP.

Autophagy is an evolutionarily conserved catalytic process by which cytoplasmic components including damaged macromolecules and organelles are degraded^43^. An increased autophagosome formation was observed in placenta from preeclampsia and FGR^44^. In this study, we found LC3-II expression was elevated in placenta from ICP. The expression of LC3-II also was increased in the placenta of ICP rat. This result indicated that autophagy was activated in ICP. Autophagy has an important role in early embryo development. Increased embryonic or fetal death caused by inactivation of autophagy related genes in mouse. Atg5 deficient mice were fertilized normally, but manifested embryonic lethality at last^45^. Beclin1 deficient mice embryos also die as early as embryonic day 7.5^46^. Previous study has demonstrated chloroquine and hydroxychloroquine inhibit bladder cancer cell growth by targeting basal autophagy^47^. Hydroxychloroquine (HCQ) inhibits autophagy and sensitize some cancer cells to chemotherapy^48^. However, as an inhibitor of autophagy, HCQ reduces neonatal morbidity in women with SLE by significantly decreasing the rate of prematurity and intrauterine growth restriction^49^. We found chloroquine (CQ) did not increase the amount of fetal mice. But induced autophagy using rapamycin, the development of fetal mice was retard and the fetuses birth weight, body length and placenta weight were decreased. Chloroquine (CQ) can rescue the retarded development and increase the fetuses birth weight, body length and placenta weight at the condition of activated autophagy. Based on these results, we guessed that activated autophagy impaired the growth of fetal mice. Inhibited autophagy promoted the growth of fetus at the condition of activated autophagy. Chloroquine is a potential drug of ICP.

To our knowledge, this is the first time reported that autophagy was activated in ICP. The expression of Linc02527 was increased in placenta and serum in ICP patients. Linc02527 bond to and stabilized YBX1, then activated P21 pathway and prompted autophagy. We also found Chloroquine (CQ) can rescue the retarded development at the condition of activated autophagy. So, Linc02527 was a potential biomarker and target for ICP. Chloroquine may be a drug for treating ICP.

## Electronic supplementary material

MS-2

MS-3

MS-4

MS-5

MS-6

MS-1

DEG_case vs control_mRNA

DEG_case vs control_lncRNA

Figure 2-S1

Figure 2-S2

S2

S3
