# Supplementary figures and images for: Linc02527 promoted autophagy in Intrahepatic cholestasis of pregnancy
Source: Cell Death Dis. 2018 Sep 24;9(10):979. doi: 10.1038/s41419-018-1013-z (PMC6155230; doi:10.1038/s41419-018-1013-z)

RT: 0.00 - 65.09

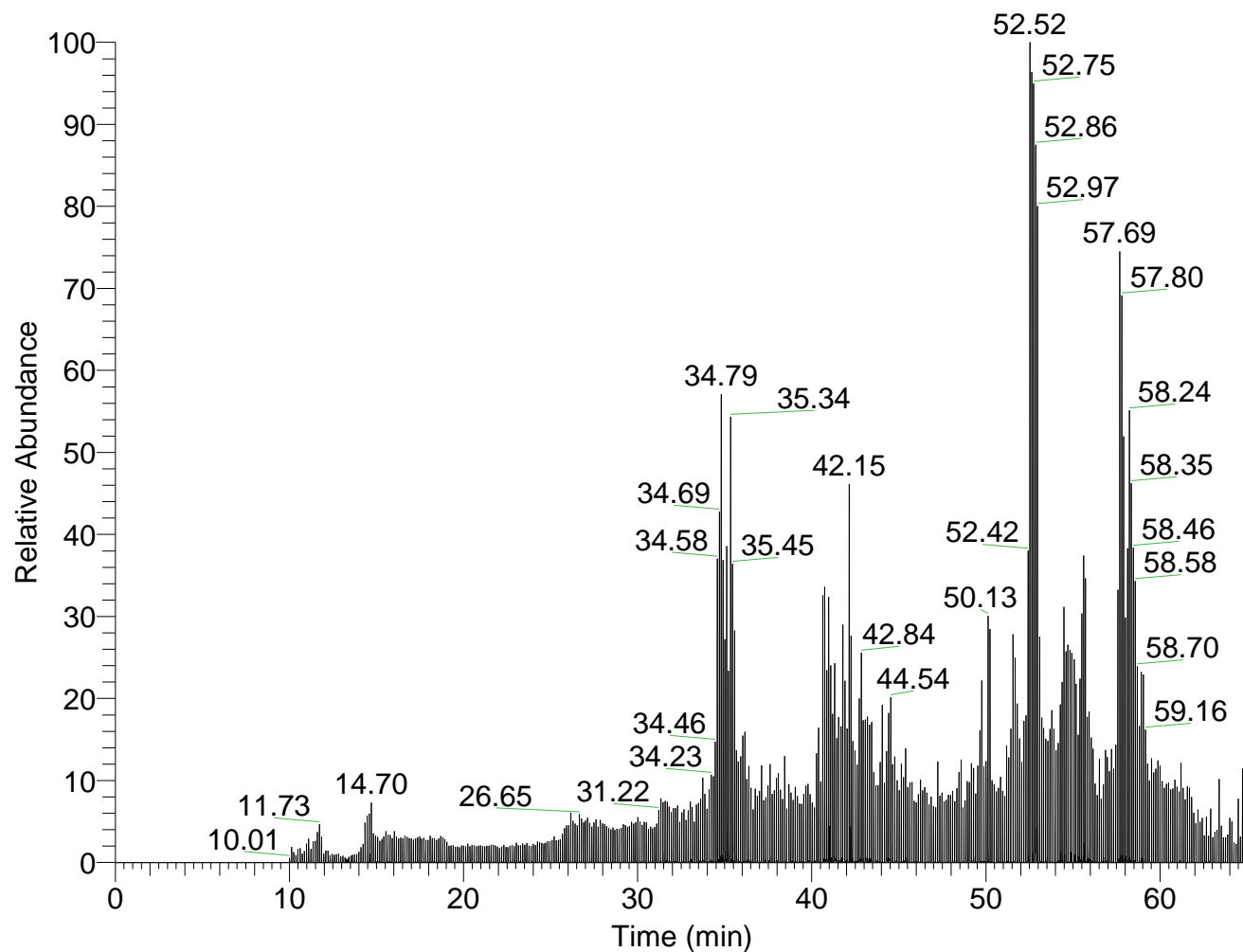

NL:  
2.10E7  
TIC MS  
GZSC\_LL\_  
B\_021517

GZSC\_LL\_B\_021517 #4892 RT: 40.02 AV: 1 NL: 9.59E1  
T: ITMS + c ESI d Full ms2 899.37@cid35.00 [235.00-2000.00]

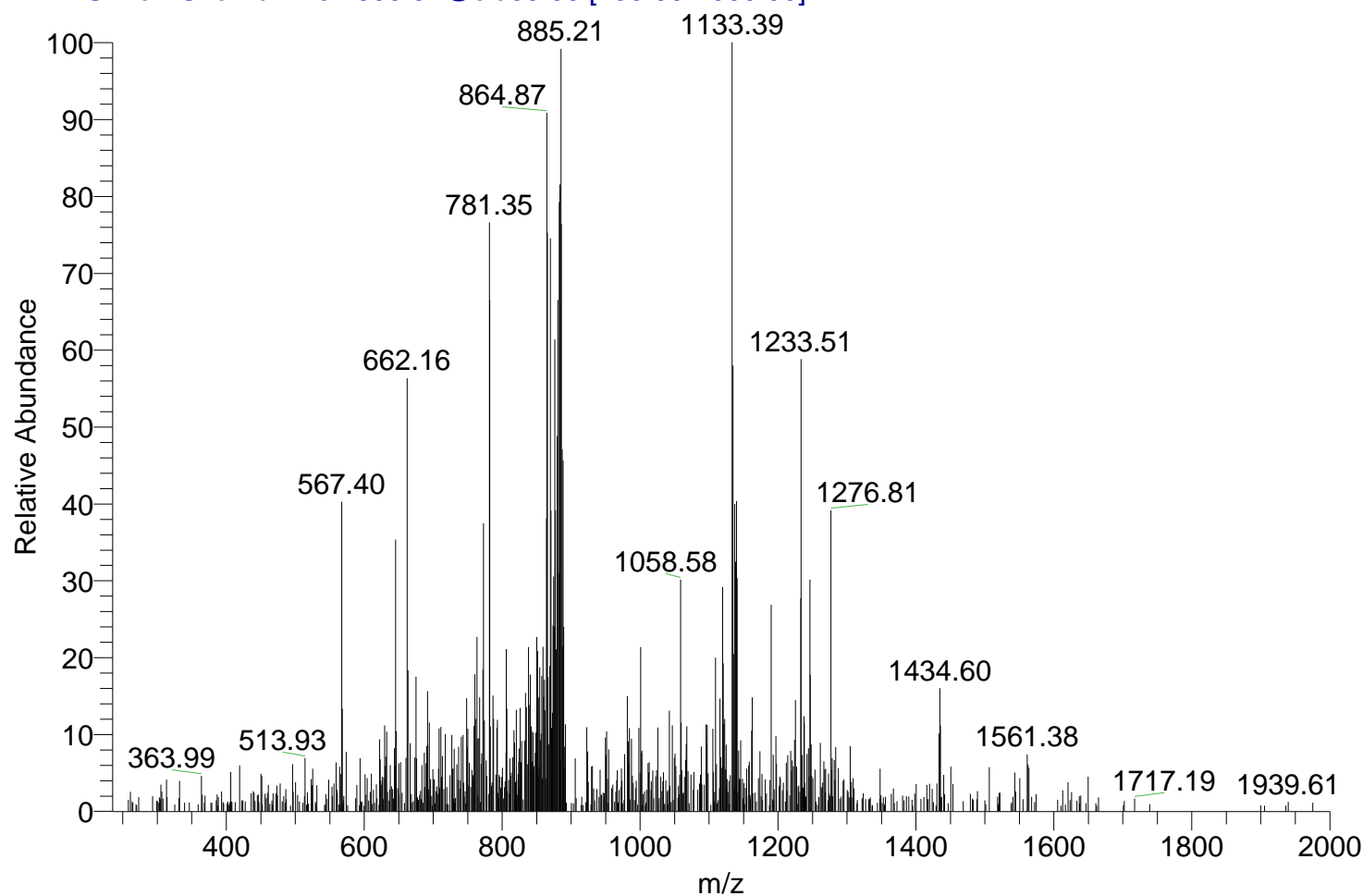

Supplement: Supplementary file 1 — MS-2 [file 41419_2018_1013_MOESM1_ESM.pdf]

RT: 0.00 - 65.00

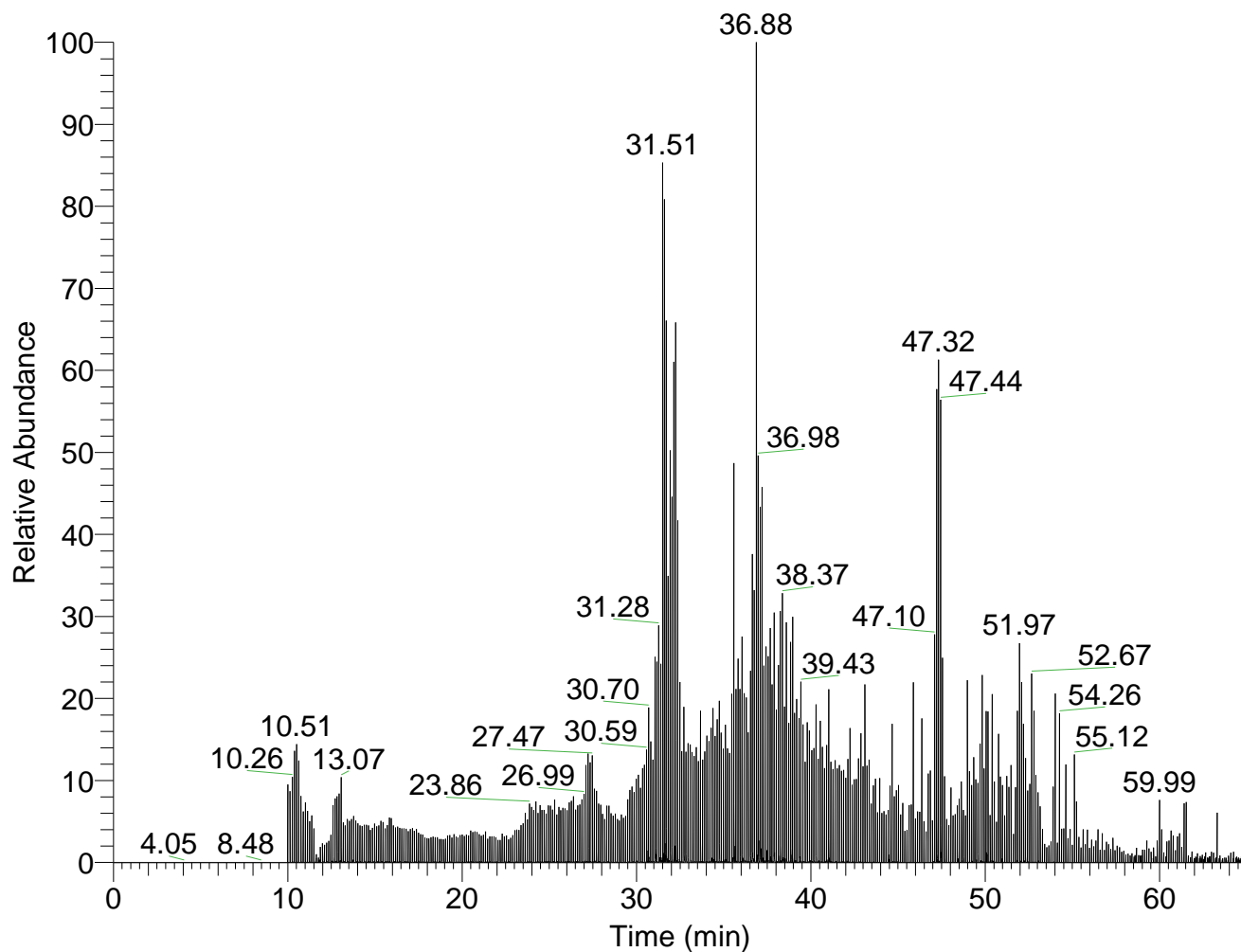

NL:  
1.63E7  
TIC MS  
GZSC\_LL\_  
C\_021417

GZSC\_LL\_C\_021417 #5985-6954 RT: 52.87-64.46 AV: 6 NL: 1.91E1

T: Average spectrum MS2 1026.23 (5985-6954)

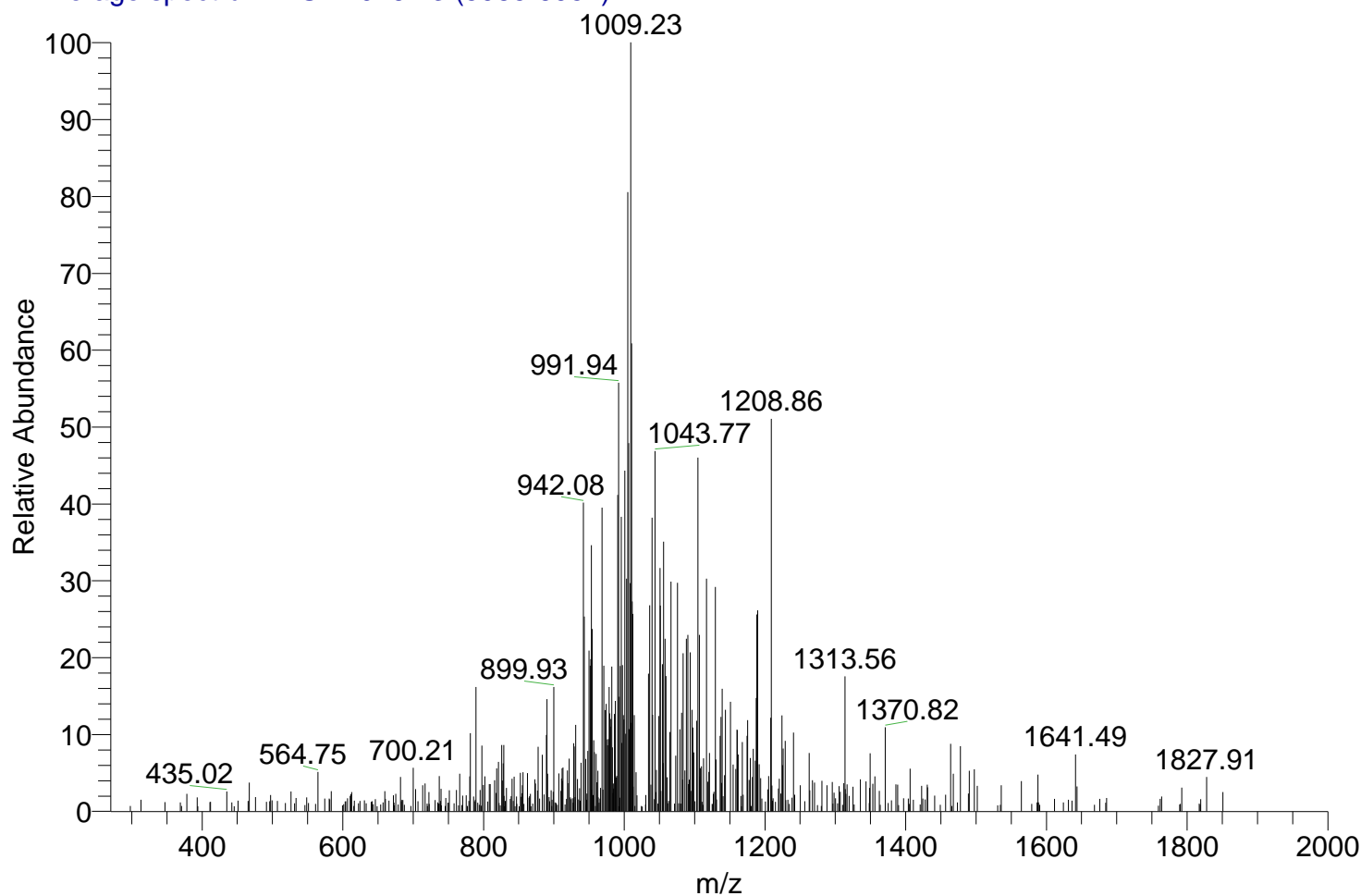

Supplement: Supplementary file 2 — MS-3 [file 41419_2018_1013_MOESM2_ESM.pdf]

RT: 0.00 - 65.00

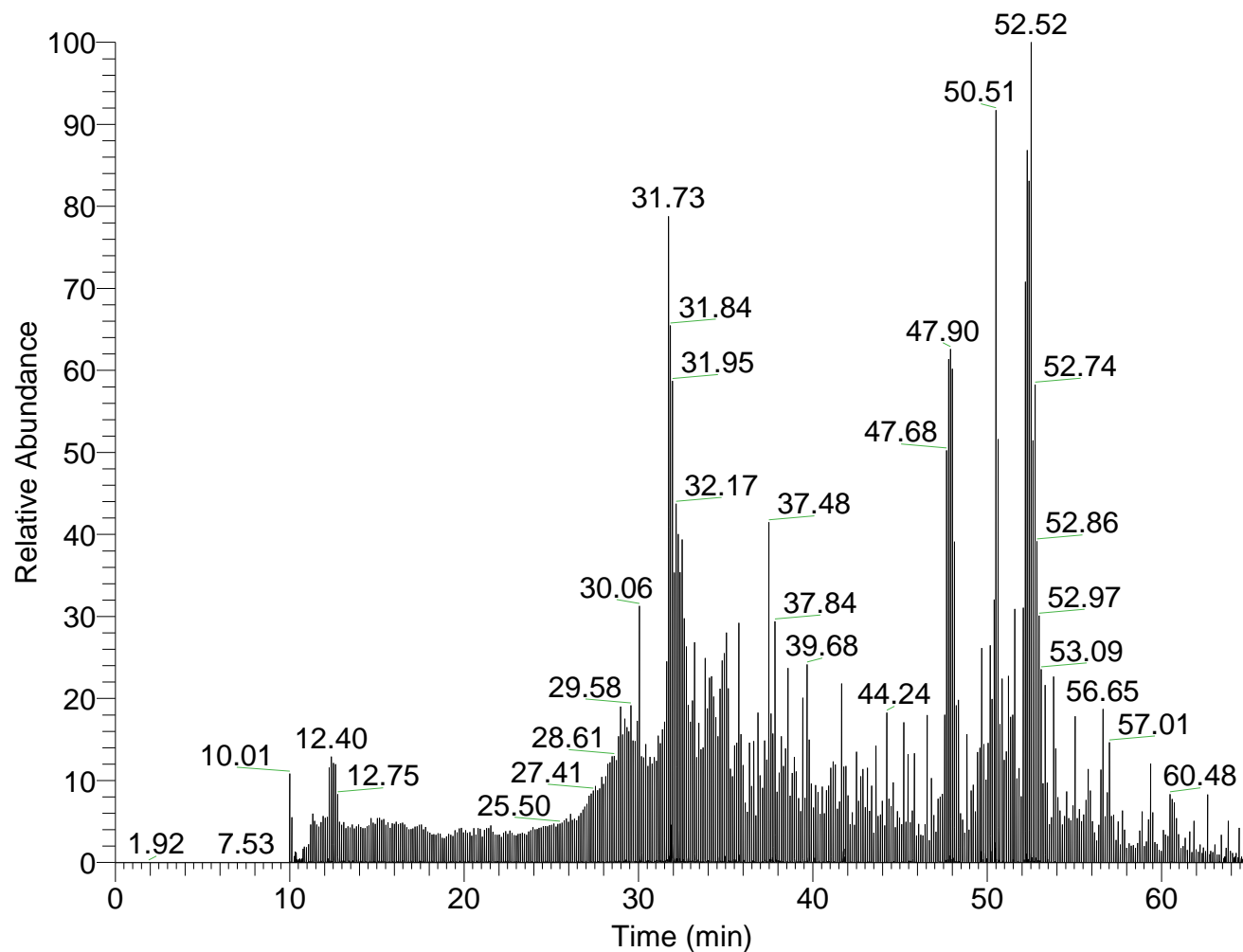

GZSC\_LL\_D\_021417 #4973-5487 RT: 40.51-46.83 AV: 3 NL: 7.57E1

T: Average spectrum MS2 918.92 (4973-5487)

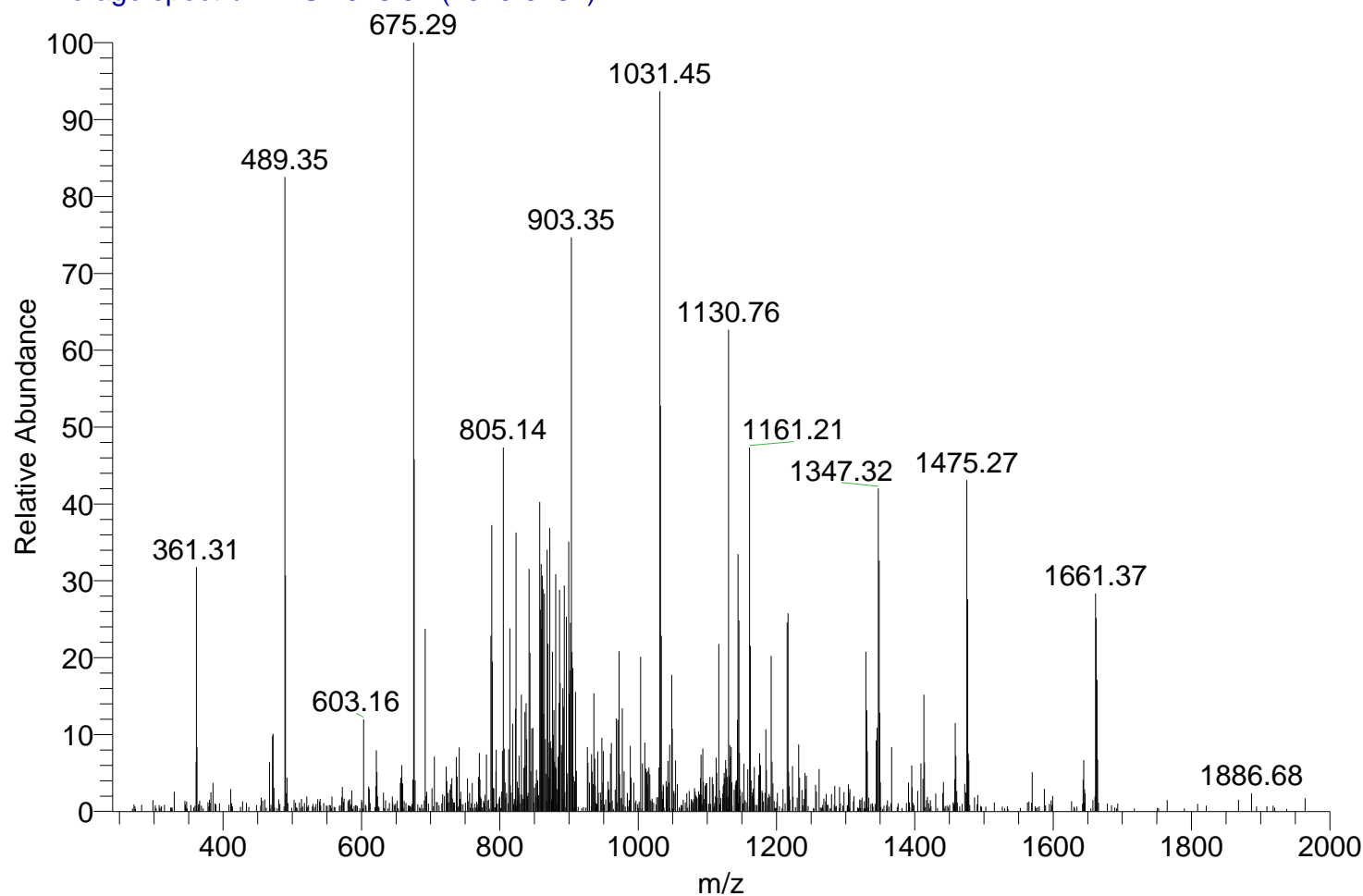

Supplement: Supplementary file 3 — MS-4 [file 41419_2018_1013_MOESM3_ESM.pdf]

RT: 0.00 - 65.04

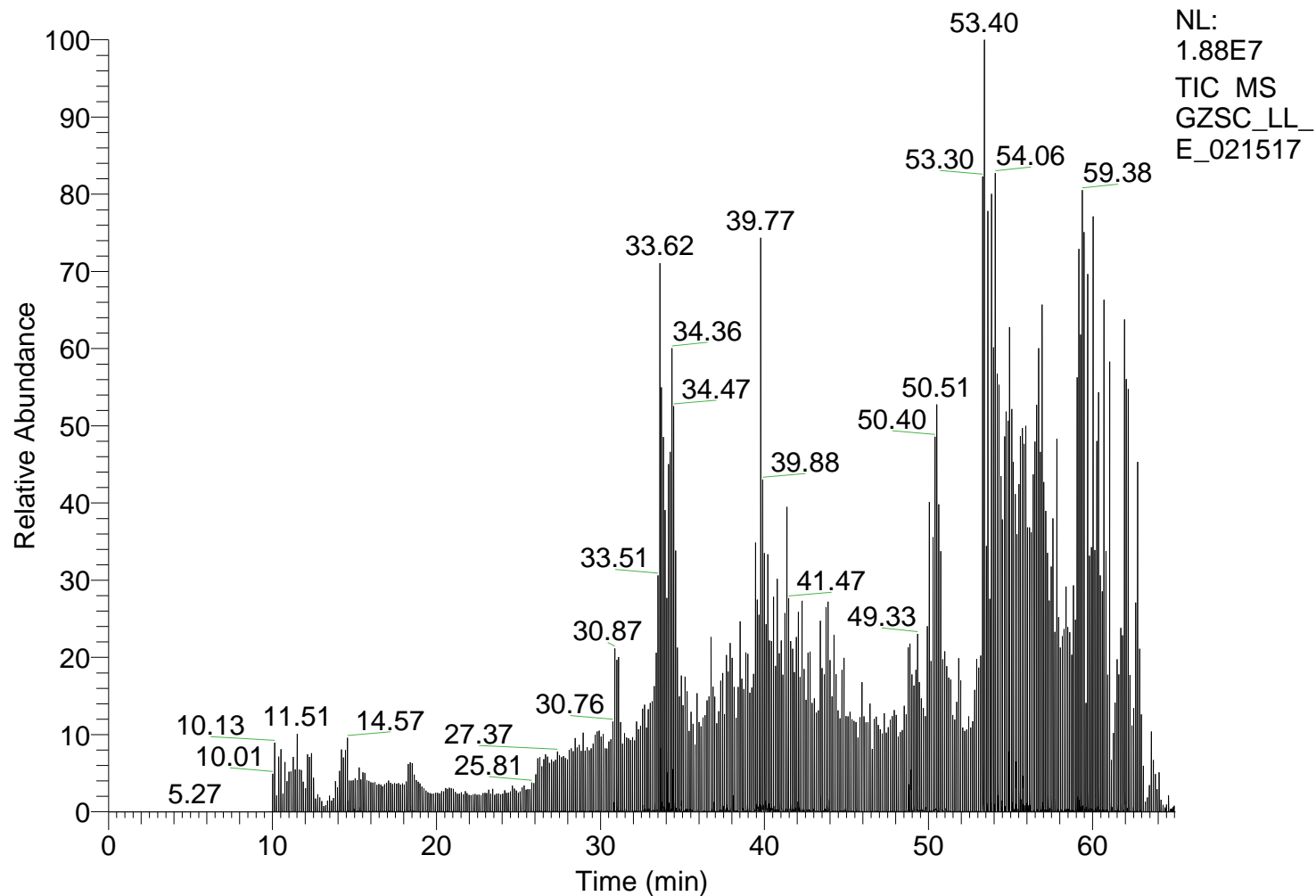

GZSC\_LL\_E\_021517 #4192-5343 RT: 31.65-45.17 AV: 2 NL: 5.28E1

T: Average spectrum MS2 965.17 (4192-5343)

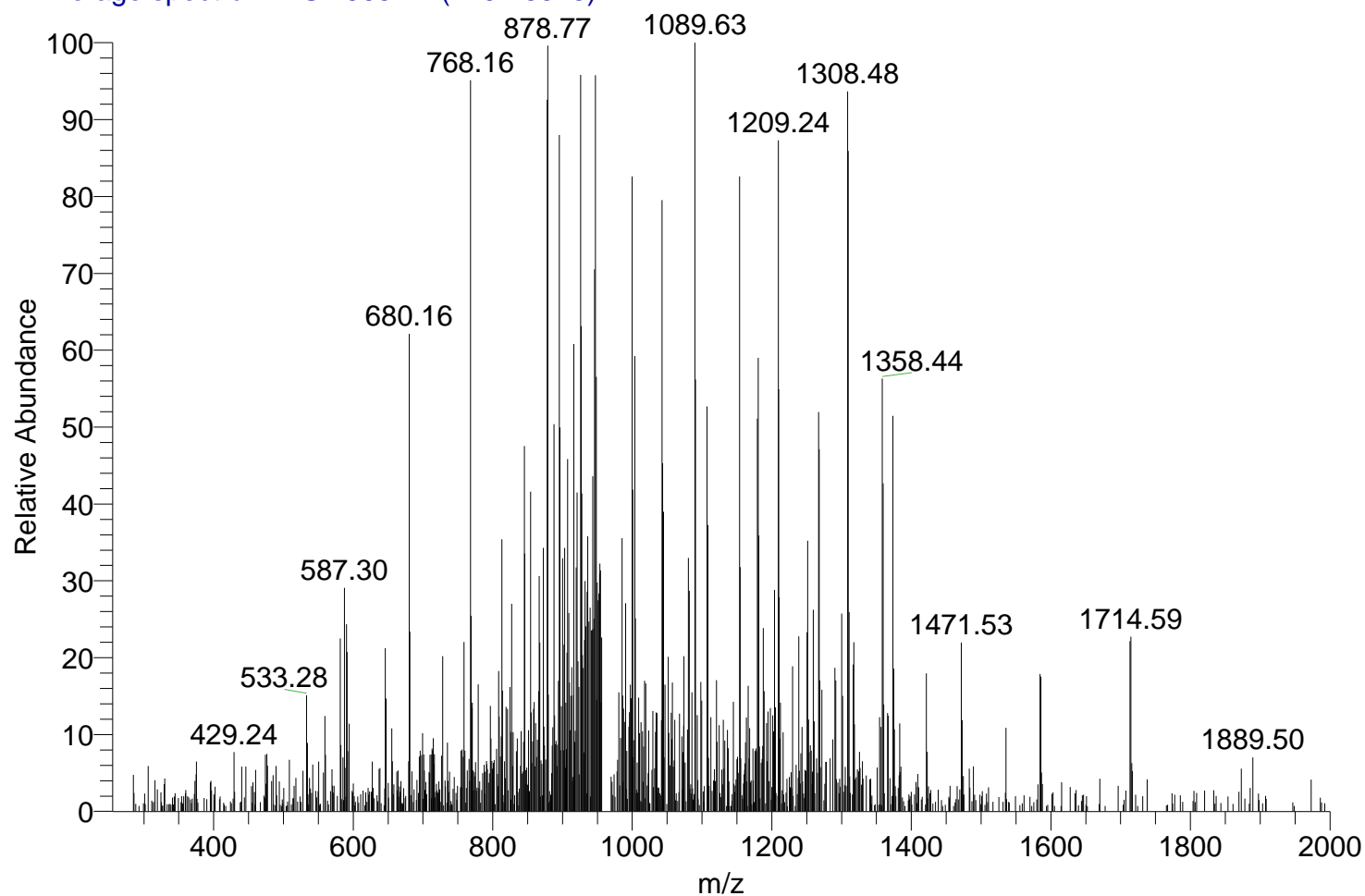

Supplement: Supplementary file 4 — MS-5 [file 41419_2018_1013_MOESM4_ESM.pdf]

RT: 0.00 - 65.08

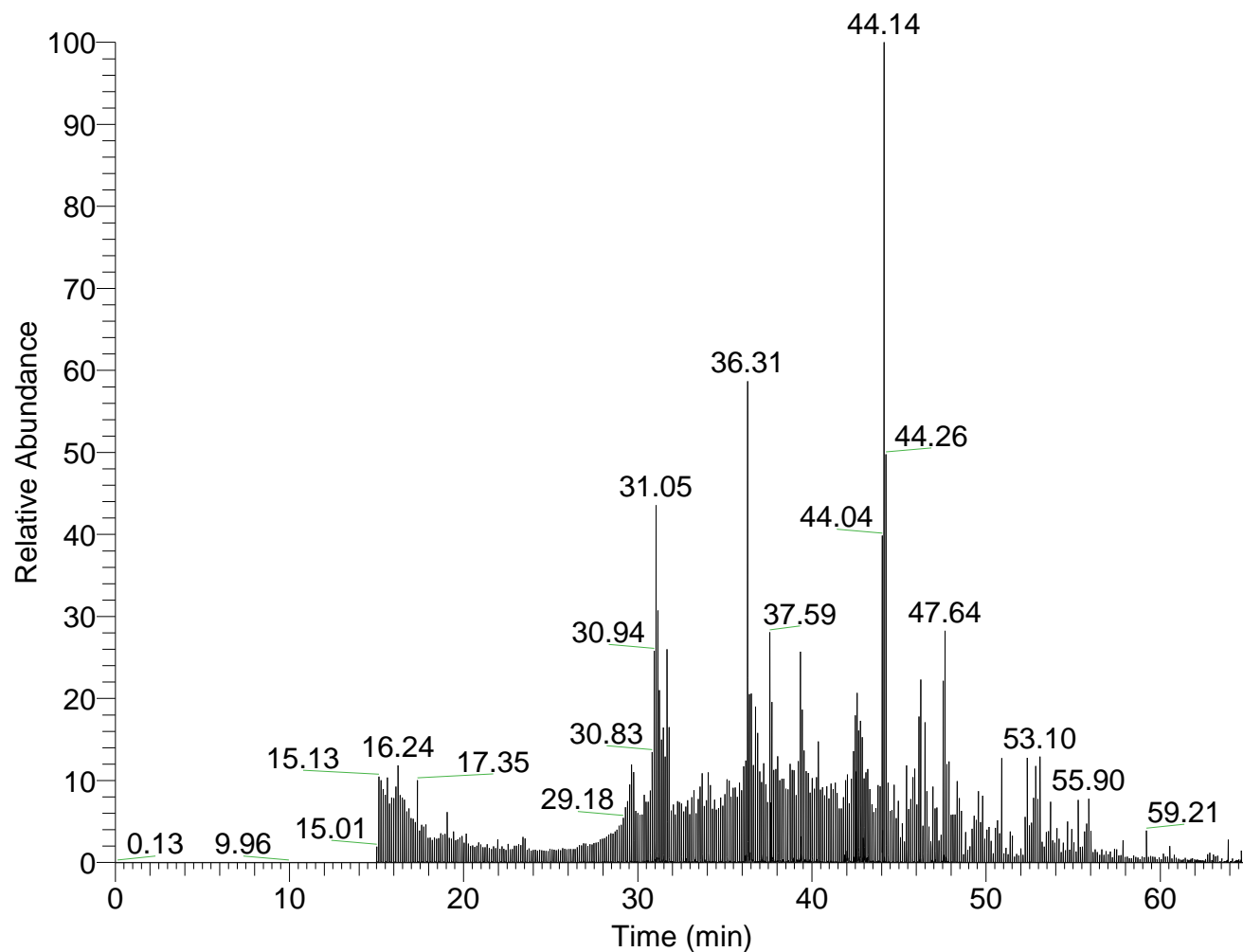

NL:  
2.97E7  
TIC MS  
GZSC\_LL-  
N\_041117

GZSC\_LL-N\_041117 #6661-7454 RT: 51.34-61.03 AV: 3 NL: 2.68E1

T: Average spectrum MS2 1069.08 (6661-7454)

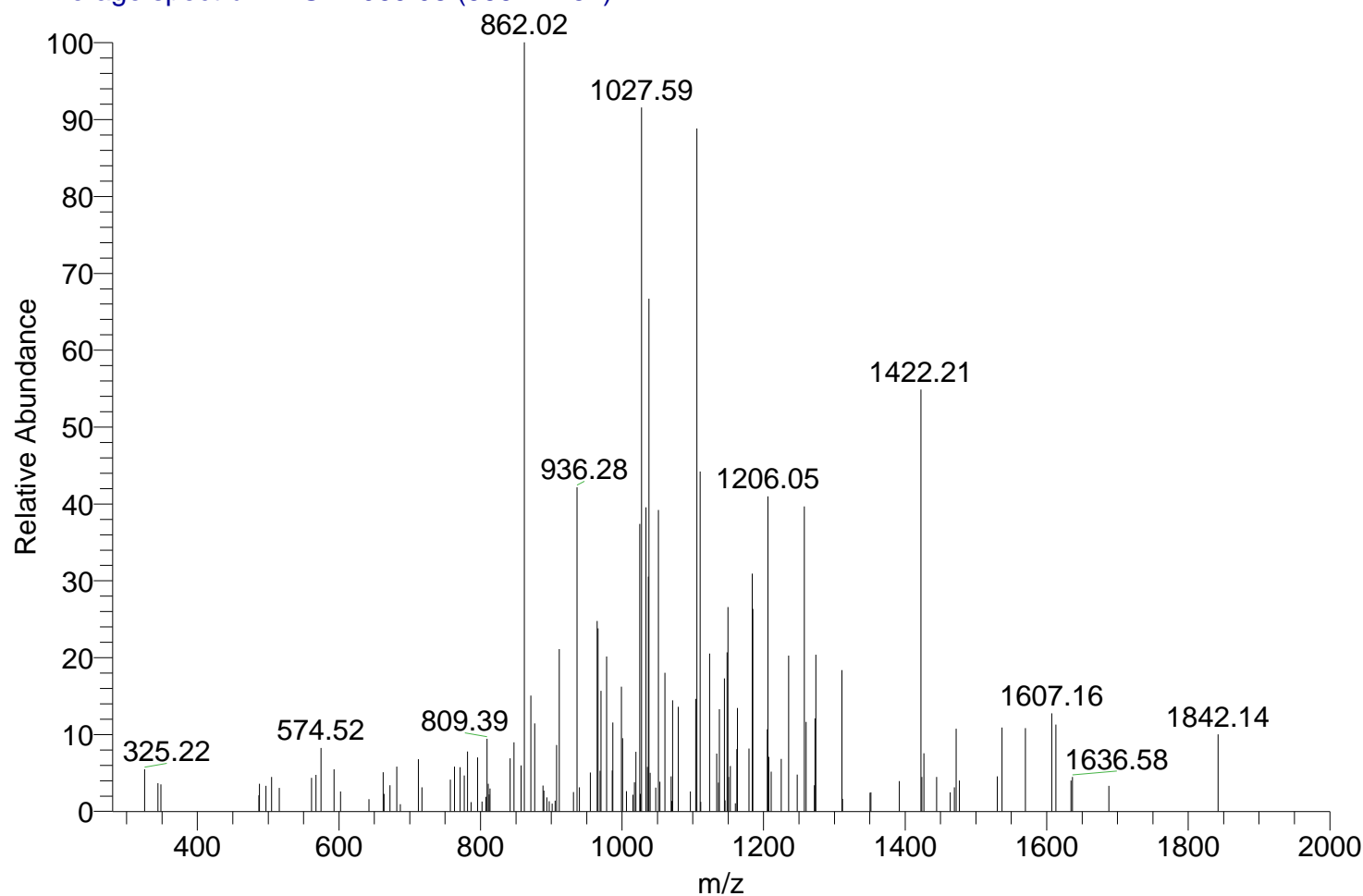

Supplement: Supplementary file 5 — MS-6 [file 41419_2018_1013_MOESM5_ESM.pdf]

RT: 0.00 - 65.06

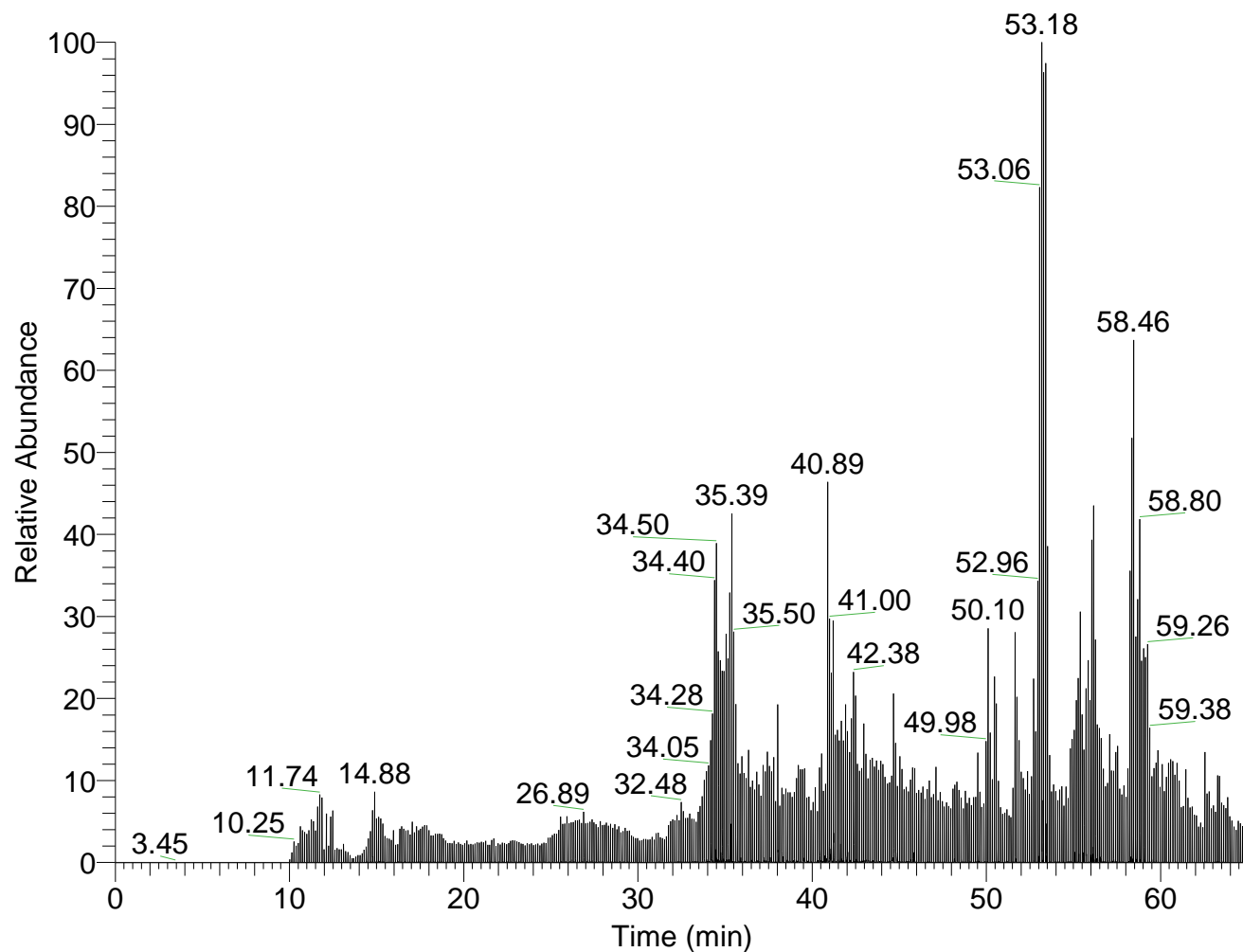

NL:  
2.18E7  
TIC MS  
GZSC\_LL\_  
A\_021517

GZSC\_LL\_A\_021517 #4940-6523 RT: 40.49-59.12 AV: 4 NL: 1.52E3

T: Average spectrum MS2 897.01 (4940-6523)

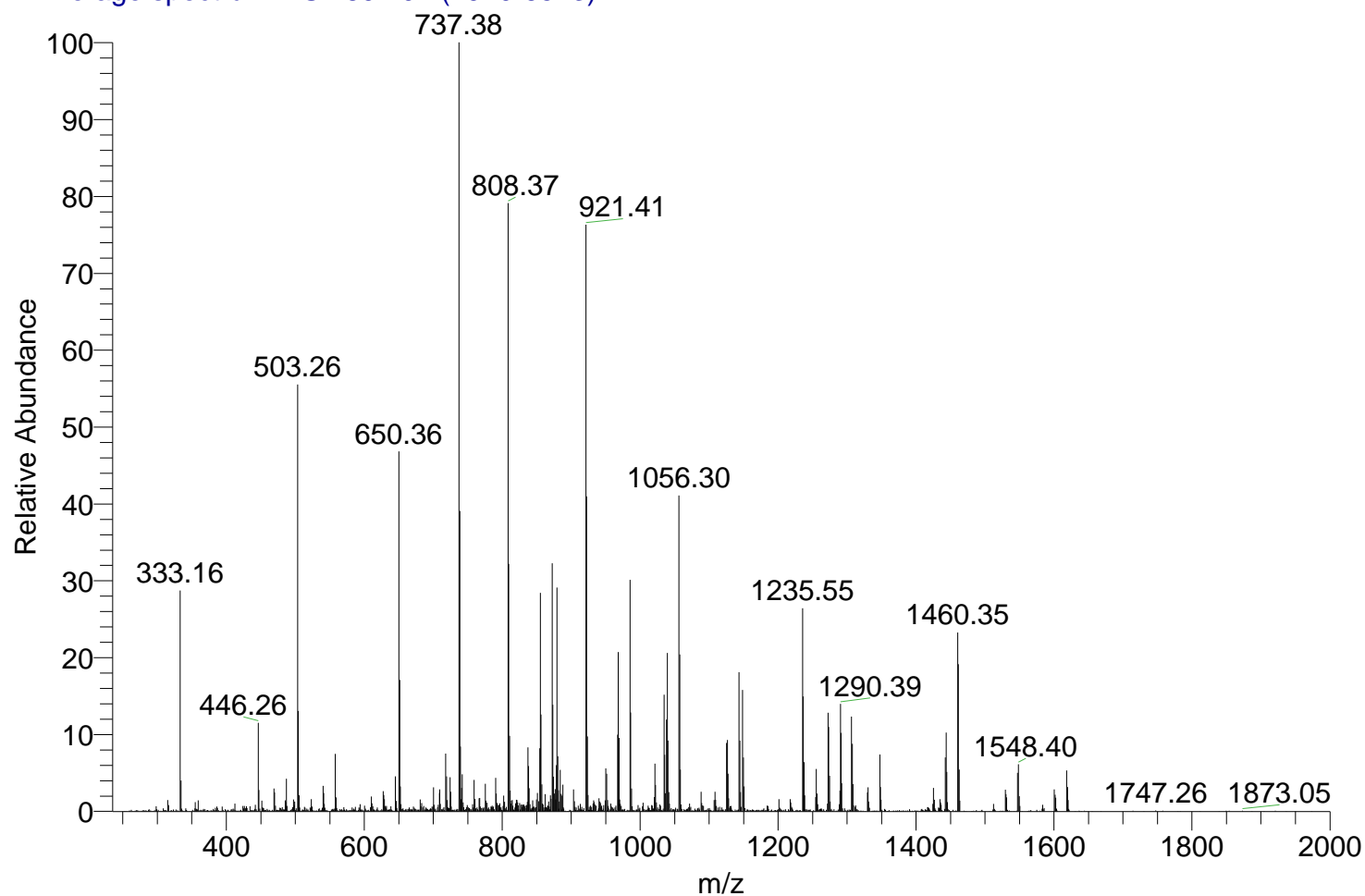

Supplement: Supplementary file 6 — MS-1 [file 41419_2018_1013_MOESM6_ESM.pdf]
